# Analysis of the need for soil moisture, salinity and temperature sensing in agriculture: a case study in Poland

**DOI:** 10.1038/s41598-021-96182-1

**Published:** 2021-08-17

**Authors:** Lech Gałęzewski, Iwona Jaskulska, Dariusz Jaskulski, Arkadiusz Lewandowski, Agnieszka Szypłowska, Andrzej Wilczek, Maciej Szczepańczyk

**Affiliations:** 1 Department of Agronomy, University of Science and Technology in Bydgoszcz, S. Kaliskiego 7, 85-796 Bydgoszcz, Poland; 2 Institute of Electronics Systems, Warsaw University of Technology, Nowowiejska 15/19, 00-665 Warsaw, Poland; 3 Institute of Agrophysics, Polish Academy of Sciences, Doświadczalna 4, 20-290 Lublin, Poland; 4 Department of Management Systems and Innovation, Lodz University of Technology, Żeromskiego 116, 90-924 Łódź, Poland

**Keywords:** Environmental sciences, Environmental social sciences, Hydrology

## Abstract

Efficient use of scarce water resources is both a marketing objective and an environmental obligation for sustainable agriculture. In modern agricultural production, which is intensive and should at the same time be environmentally friendly, there is a need to monitor soil moisture, salinity and temperature. The aim of the study was to determine the demand of producers of agricultural and horticultural plants for equipment and systems for monitoring soil properties at an individual farm level in regions with highly developed agriculture. A questionnaire survey was conducted among 1087 respondents, also direct interviews in Poland were undertaken. According to the producers' responses, it is important to know soil moisture, salinity and temperature, although currently only about 4% of the surveyed farmers have the equipment to evaluate these soil parameters. In their view cost is not the most important obstacle to the purchase of the necessary probes. More important is that the devices should be easy to install and use, and have an easy to use application for data collection, processing and transfer. The current market does not offer solutions that meet these producers expectations. The demand for suitable probes is very high as over 80% of the farmers declared their willingness to purchase such probes. Technical problems related to the operation and servicing of such equipment were the most frequently mentioned impediments in their use. However, farmers and horticulturists believe that knowledge of their soil properties would allow them to optimize the elements of cultivation technology, including the use of plant irrigation systems, the use of mineral fertilizers and plant protection products.

Climatic conditions shape plant production- not only the yields, but also the area sown, production intensity and selection of technologies^1^. Climatic elements, especially the rainfall and the air temperature are necessary for the proper functioning of the physiological processes of crops^2^, their development and productivity^3^, as well as for shaping the broadly understood properties of soil^4^. Crop plants are sensitive to climate change: deficiency or excess water, or suboptimal temperatures may cause severe abiotic stresses^5,6^. Drought stress causes molecular, biochemical, physiological and morphological changes in plants^7^. Plants then grow smaller organs, e.g. roots, leaves, and also their productivity, biomass and yield are lower^8^. According to Daryanto et al.^9^, a shortage of about 40% of water in relation to the needs of wheat and maize may reduce the yield of these plants by around 20% and 39%, respectively. The results of research conducted on a global scale also indicate the negative impact of an increase in temperature on the yields of the crops most important for feeding humanity. An increase in temperature by one degree Celsius reduces the yield of maize by 7.4%, wheat by 6.0%, rice by 3.2% and soy by 3.1%^10^.

Climate change, limited water resources and the high demand for water in agriculture require an increase in the efficiency of water use^11,12^. This can be possible through interdisciplinary research and activities in the field of plant genetics and biology, as well as in measurement technology and agrotechnology^13,14^. The first condition is the monitoring of rainfall and air temperature and other climatic elements that strongly affect the water balance in the soil available to plants^15,16^. There are many sources of data on that topic in the literature^17–19^, however, they are often not very accurate and vary in terms of their measurement methodology^20^. The spatial and temporal monitoring of soil moisture is even more difficult. There are many methods for making direct and indirect evaluations of soil moisture: thermogravimetric direct method, electrometric method, capacitance method, frequency domain reflectometry, time domain reflectometry and the neutron method^21,22^. However, there are few systems for monitoring and collecting data that use a standardized method for soil moisture measurements, as well as for probe data registration, collection and transmission from multiple stations located over a large area. One example of such a system is the International Soil Moisture Network (ISMN) that collects data on soil moisture world-wide^23^.

The lack of accurate information concerning the spatial differentiation of soil water resources available to plants greatly limits the effectiveness and economic legitimacy of sustainable and precision agriculture. The idea of precision agriculture is the application of production means in accordance with the conditions resulting from soil variability, including its moisture, temperature and salinity^24,25^.

In regions with highly developed agriculture and horticulture, producers eagerly use the benefits of technical and technological progress, although in the case of precision agriculture their implementation in Europe is smaller than it is in American or Australian agriculture^26^. In the literature, however, there are few reports on the practical use of devices and systems for the monitoring of spatial and temporal variability of soil properties at a field or farm level. Also, not all modern technologies are accepted among producers, which may be due to a lack of confidence in their effectiveness^12,27,28^. It was thus hypothesized that despite the large market demand for measurement solutions, their popularization in agricultural and horticultural production would require universal, durable, technically uncomplicated, easy-to-use and inexpensive devices. Such requirements can be met by dielectric probes. The knowledge on soil moisture, temperature and salinity is needed by producers to make many decisions regarding not only plant irrigation^29,30^, but also for optimization of fertilization, as well as of dates and methods of tillage, sowing, cultivation measures and plant harvesting.

The purpose of the present work was to determine the demand of producers of agricultural and horticultural plants in regions with highly developed agriculture for devices and monitoring systems of soil properties at the farm level. The research was aimed at understanding the expectations of farmers and horticulturists regarding the functional features of such solutions and their expected usefulness in making production decisions. A critical analysis of the global market of available and easy-to-use dielectric solutions for assessing the continuous changes in soil properties during the vegetation period was also performed.

## Material and methods

**Figure 1:**
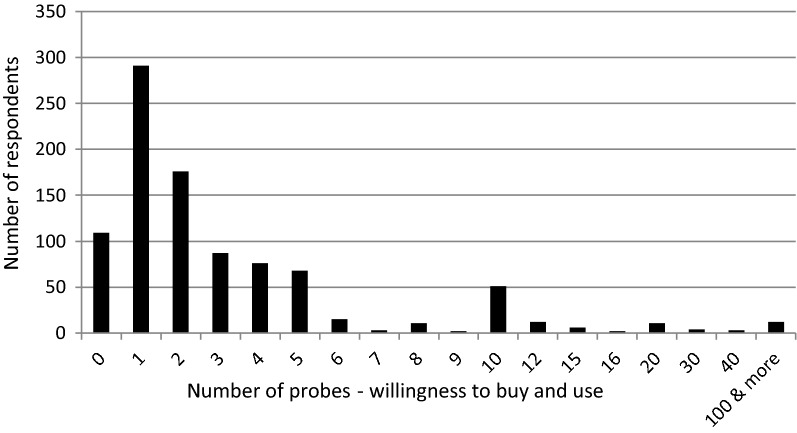
Number of respondents declaring willingness to buy and use a certain number of probes.

**Figure 2:**
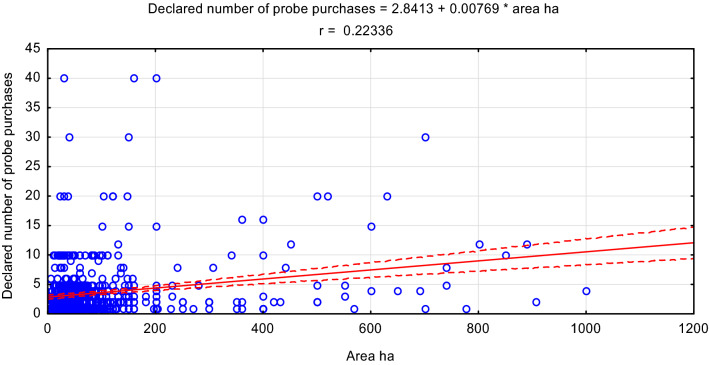
Relationship between the size of the farm and the declared number of purchases and use of probes.

**Figure 3:**
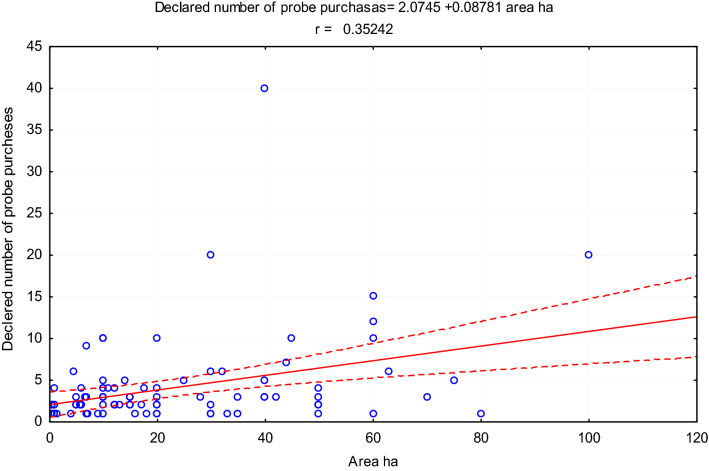
Relationship between irrigated area on the farm and the declared number of purchase and use of probes.

**Figure 4:**
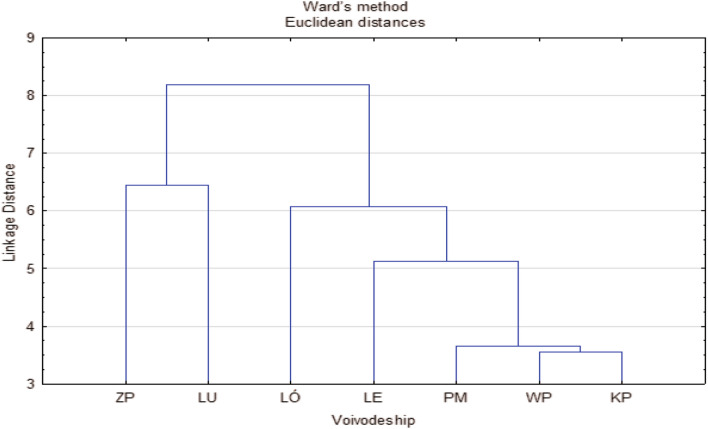
Clusters of voivodeships with similar farmers' expectations regarding the features of probes for the assessment of soil moisture and salinity. Voivodeship: KP-Kuyavian-Pomeranian, LE-Lublin, LU-Lubusz, LÓ-Łódź, PM-Pomeranian, WP-Greater Poland, ZP-West Pomeranian.

**Figure 5:**
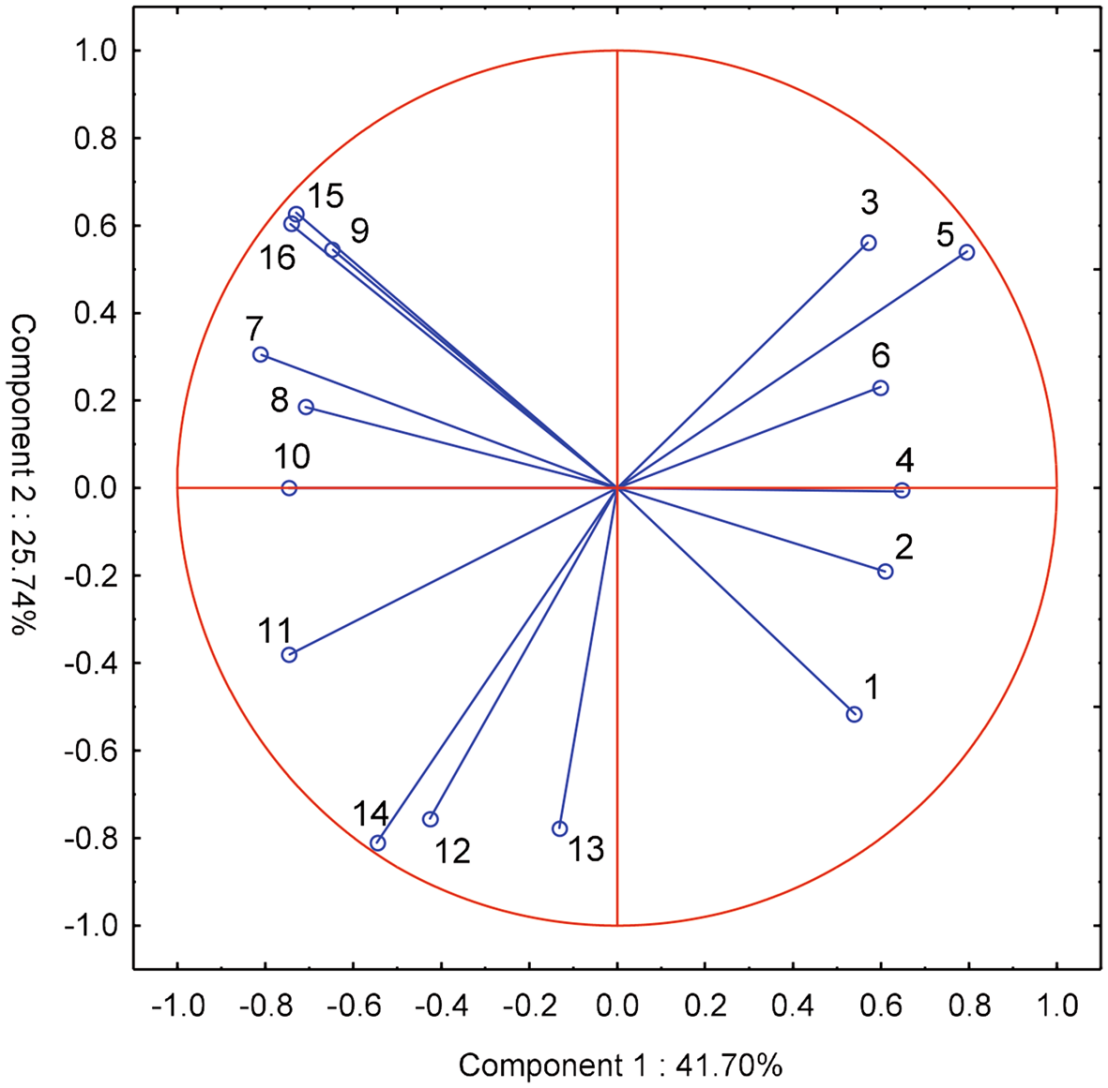
Main components of farmers' expectations in voivodeships with regard to the possibility of using probes to optimize agrotechnical treatments: 1-date of commencement of field works, 2-sowing date, 3-sowing depth, 4-method of soil cultivation, 5-method and dose of fertilization, 6-application of plant protection products, 7-dates of fertilization. With regard to performance characteristics of the probes: 8-price, 9-measurement accuracy, 10-measurement range 0–15 cm, 11-measurement range 0–30 cm, 12-reliability and resistance to damage, 13-easy installation and operation, 14-sending results to a smartphone/computer, 15-information about the dose of water for irrigation, 16-measurement data used to automate irrigation.

**Figure 6:**
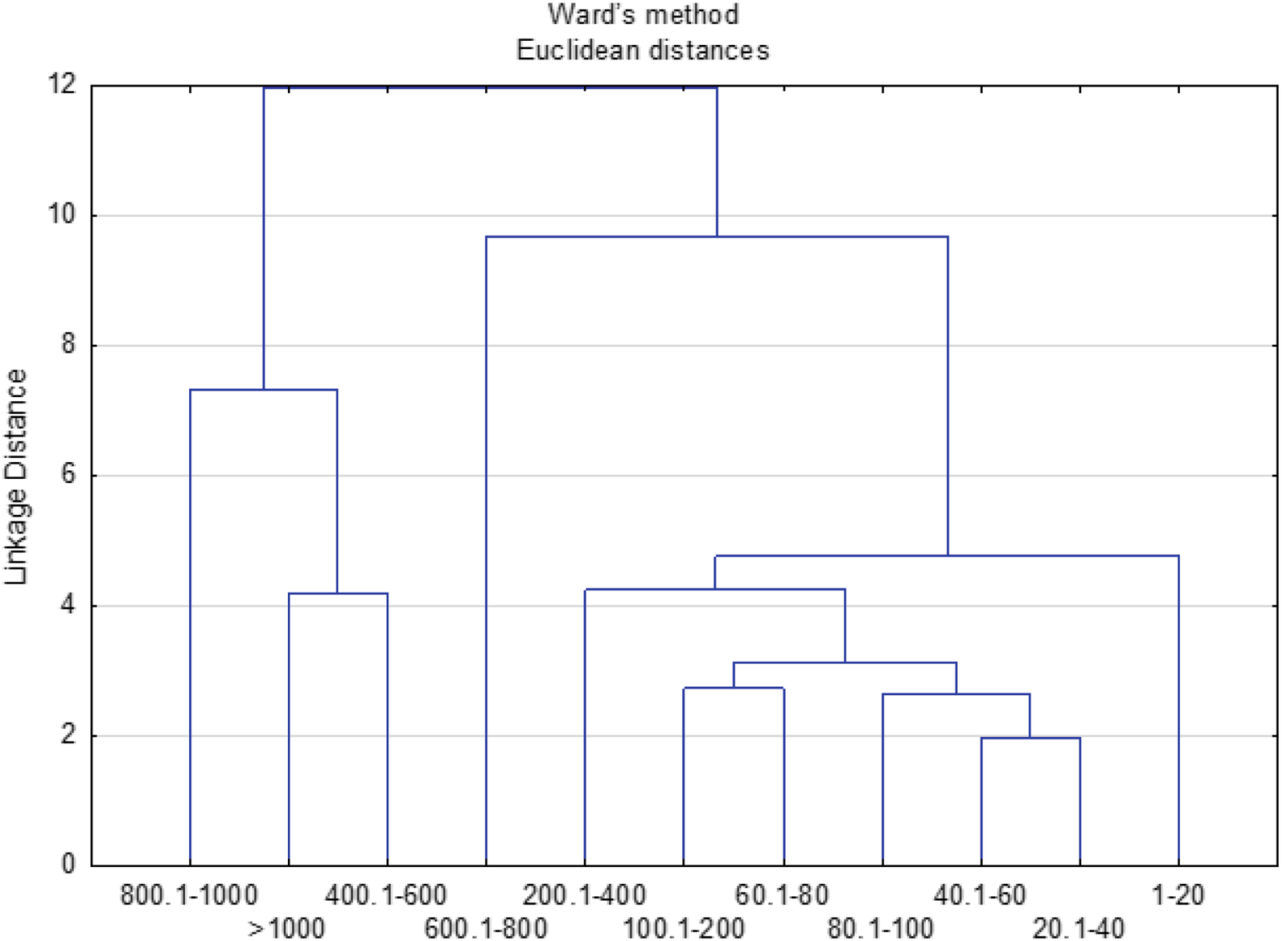
Clusters of farms of different areas (ha) with similar expectations of farmers in terms of the features of the soil moisture and salinity probes.

**Figure 7:**
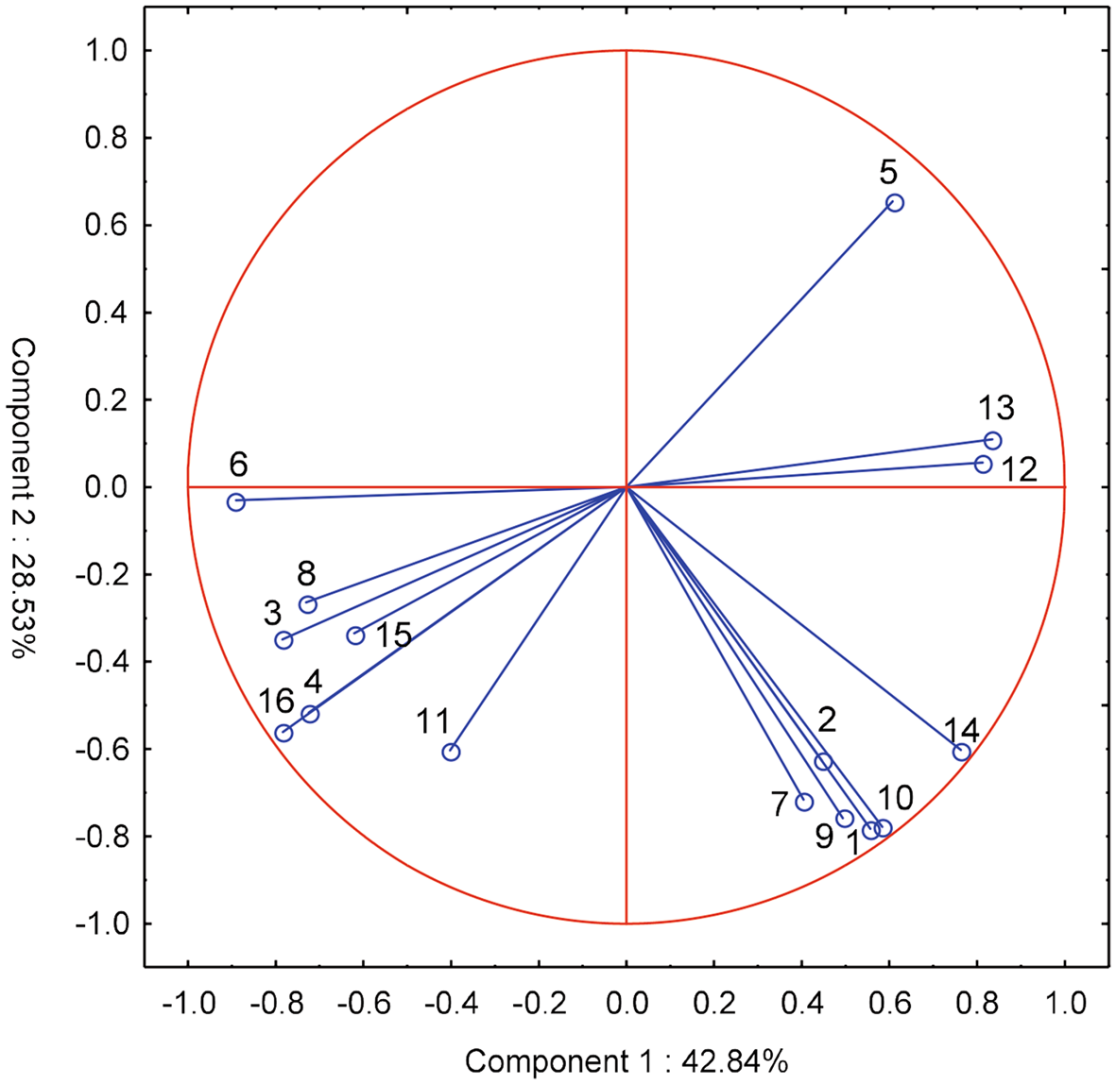
Main components of expectations of owners of farms with different areas with regard to the possibility of using probes to optimize agrotechnical treatments: 1-date of commencement of field works, 2-date of sowing, 3-sowing depth, 4-method of soil cultivation, 5-method and fertilization dose, 6-application of plant protection products, 7-dates of fertilization. With regard to functional features of the probes: 8-price, 9-measurement accuracy, 10-measurement range 0–15 cm, 11-measurement range 0–30 cm, 12-reliability and resistance to damage, 13-easy installation and operation, 14-sending results to a smartphone/computer, 15-information on the dose of water for irrigation, 16-measurement data used to automate irrigation.

**Figure 8:**
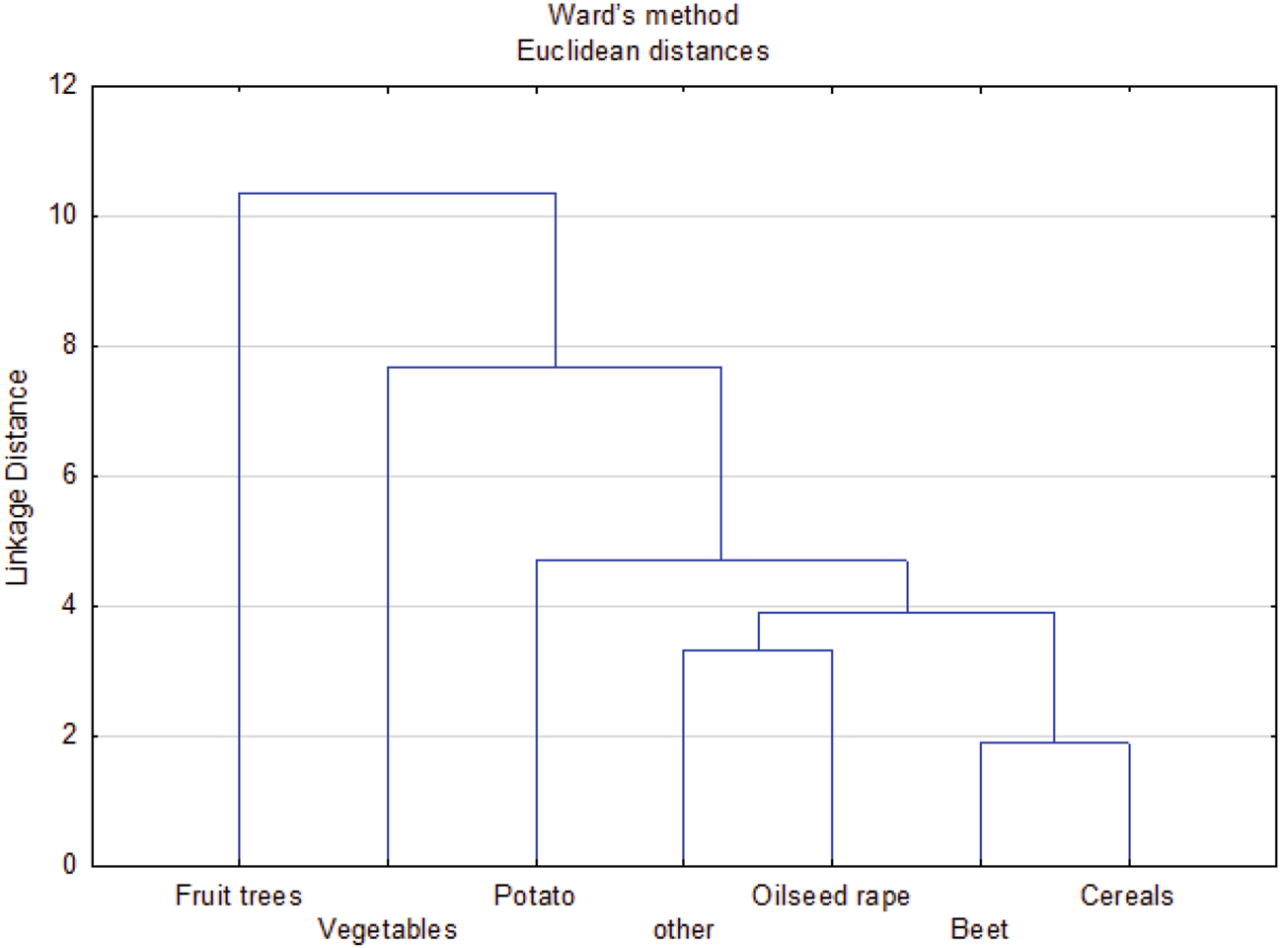
Clusters of groups of plants cultivated by farmers with similar expectations regarding the features of soil moisture and salinity probes.

**Figure 9:**
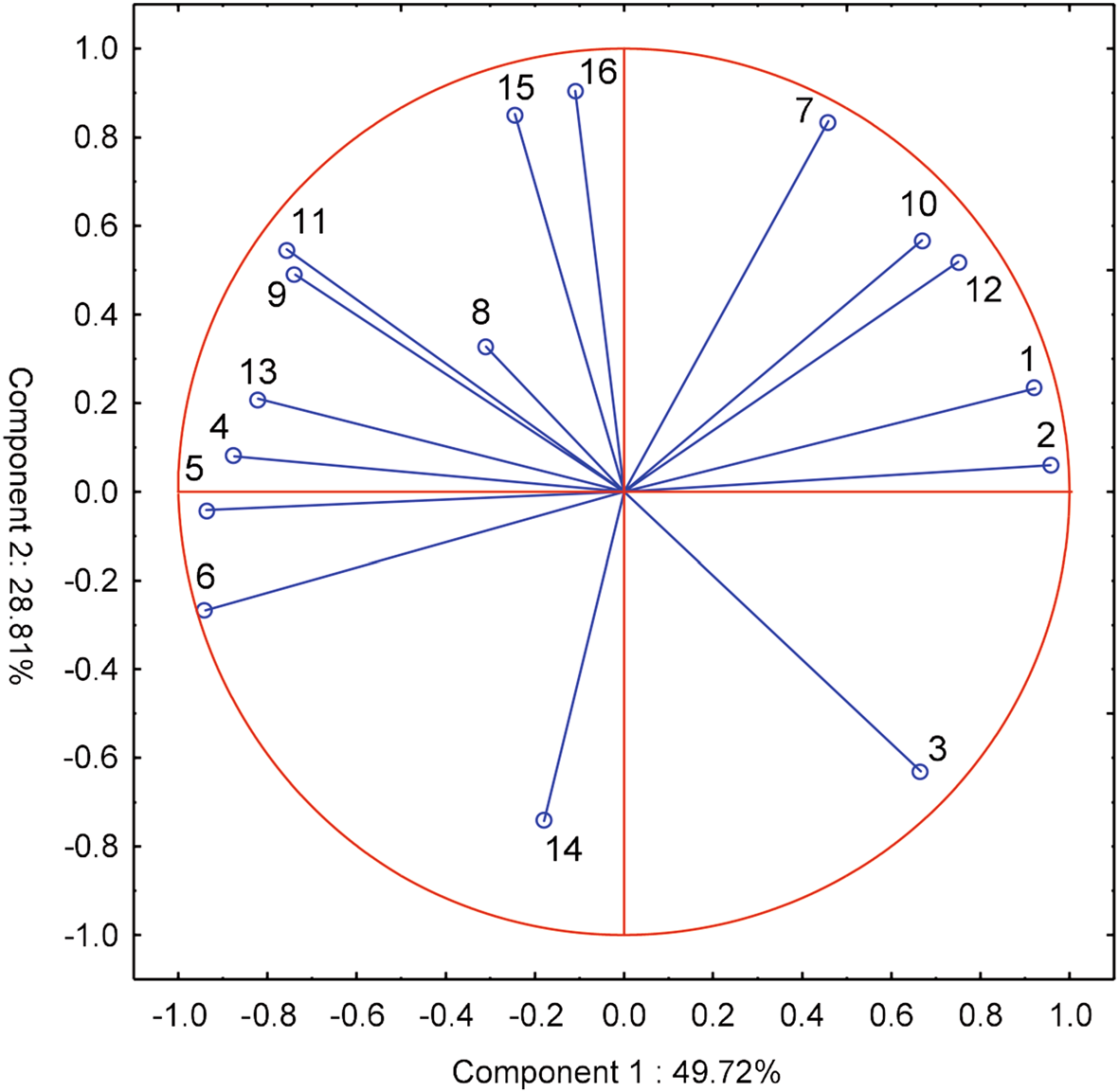
Main components of the expectations of farmers cultivating various groups of plants with regard to the possibility of using probes to optimize agrotechnical treatments: 1-date of commencement of field works, 2-sowing date, 3-sowing depth, 4-soil cultivation, 5-method and fertilization dose, 6-application of plant protection products, 7-dates of fertilization. With regard to functional features of the probes: 8-price, 9-measurement accuracy, 10-measurement range 0–15 cm, 11-measurement range 0–30 cm, 12-reliability and resistance to damage, 13-easy installation and operation, 14-sending results to a smartphone/computer, 15-information about the dose of water for irrigation, 16-measurement data used to automate irrigation.

The research was carried out in the following stages:

- Formulation of the research problem
- Research method—survey preparation
- Surveying the farmers
- Characterizing respondents' farms (location, acreage, crop structure)– Tables 1, 2, 3
- Assessment of farmers' (respondents) knowledge about the need for and possibilities of soil properties monitoring – Tables 4, 8
- A review of the state-of-the-art in relation to probes for assessing moisture, temperature and salinity of soils – Table 5
- Analysis of the needs and expectations of farmers in relation to the probes depending on the actual farming conditions– Tables 6, 7, 9, Figs. 1, 2, 3
- Multivariate analysis of the features that farmers would expect from soil properties monitoring probes’– Figs. 4, 5, 6, 7, 8, 9

The source material consisted of the results of the survey and face-to-face interviews. The respondents consisted of owners or managers of farms in Poland. The respondents were selected randomly during the most important national agricultural meetings in the summer of 2018. 1087 correctly completed questionnaires were obtained, which is a representative sample. The respondents had farms located in 246 communes in 10 voivodeships (provinces) that have the most developed agricultural and horticultural production in Poland. The selection of the research area, the diversity of respondents and the economic potential of their farms enables a generalization of the obtained results to many agricultural regions in Europe and in the world.

The survey questionnaire and the interview scenario included questions about the location of the farm, its area, the species structure of cultivated plants and the use of crop irrigation. The questionnaire also included questions about the farmers’ knowledge of the impact of soil properties on the applicability and effectiveness of cultivation elements. The resulting data was subjected to mathematical and statistical analysis. By grouping results according to a given criterion, incorrectly filled questionnaires were removed. Excel 2016 spreadsheet (Microsoft Corporation) and the statistical package Statistica 12.5 (StatSoft Inc.) were used to analyse the results. Multivariate analyzes were also performed. The results of the surveys and interviews were analyzed in three groups of respondents: territorial - voivodship, area - farm size, and crop structure - species and groups of crops. In the first group, there were 7 cases (with over 10 respondents in each voivodeship), in the second group-11 cases, and in the third - 7 cases. The variables were the functional and technical features (16 traits) of probes for monitoring soil moisture and salinity as well as the agronomic functionalities of these probes that were expected by farmers to be useful for optimization of plant production. The percentages of respondents' positive responses were treated as values for individual features and were standardized prior to cluster analysis and principal component analysis. The result of the cluster analysis is presented as a dendrogram after using Ward's method for grouping cases. The results of the principal component analysis (two principal components) are presented as a projection of primary variables on the plane.

### Characteristics of the surveyed population

The largest number of surveys were carried out in regions with well-developed field agricultural and horticultural production—vegetable growing, fruit growing, i.e. in Kuyavian-Pomeranian voivodship - 55.0% of surveys, Greater Poland - 23.4%, Pomeranian—9.8%, as well as in West Pomeranian, Lublin, Lubusz and Łódź—each more than 1% (Table 1).

**Table 1:** Geographical and administrative structure of the survey area.

Voivodeship	Number of communes	Respondents	
		Number	%
Lower Silesian	2	5	0.46
Kuyavian-Pomeranian	88	595	55.04
Lublin	23	26	2.41
Lubusz	8	22	2.04
Łódź	8	16	1.48
Masovian	7	8	0.74
Pomeranian	31	106	9.81
Warmian-Masurian	5	9	0.83
Greater Poland	47	253	23.40
West Pomeranian	26	41	3.79
Total	245	1081	100.00

The surveys covered farms diversified in terms of their area, the type of agricultural land and the crop structure. The test sample included both small farms and large agricultural enterprises with several thousand of ha of agricultural land. The largest group was family farms with an area of 20–40 ha, which represented close to 30% of the tested sample (Table 2). The percentage of large (> 100 ha) and very large (> 1000 ha) farms was higher than it would have been if based on their share in the area structure of agricultural holdings in Poland. This is a consequence of the data collection methodology. The survey questionnaire and direct interviews were carried out mainly among the owners and users of farms with a high production potential and, to a lesser extent, in small farms producing for self-supply of the family and not having a commercial character. On the other hand, such a disproportion in the surveyed area groups of farms is justified, since farms with larger areas constitute more of a potential market for devices for monitoring the soil moisture, temperature and salinity.

**Table 2:** Area structure of the examined agricultural and horticultural farms.

Farm area, ha	Proportion	
	Number	%
1–20	225	22.0
20.1–40	304	29.7
40.1–60	155	15.1
60.1–80	98	9.6
80.1–100	67	6.5
100.1–200	102	10.0
200.1–400	31	3.0
400.1–600	17	1.7
600.1–800	10	1.0
800.1–1000	5	0.5
> 1000	11	1.1
Total	1025	100.0

A particularly important group of the respondents interested in soil moisture monitoring were the owners of farms who grow commodity crops while applying irrigation. Thus, farms were grouped based on the presence (Yes, No) of irrigation systems and cultivated plants. Only 11 farms (1.01%) did not grow basic cereals. These were farms specialized in the cultivation of vegetables, maize or other crops (Table 3). Irrigation was used mainly in horticultural crops—in vegetables—on 29% of farms, orchards - 44% of farms and potatoes - 17%. In Poland, however, farms cultivating basic agricultural crops, such as cereals, oilseed rape and sugar beet, dominate. In the study group, these crops constituted 99.0%, 56.5% and 69.9% of holdings, respectively, and about 10% of them apply irrigation.

**Table 3:** Crop structure and plant irrigation on agricultural and horticultural farms.

Crops	Irrigation	Presence			
		Yes		No	
		Number	%	Number	%
Cereals	No	960	89	8	73
	Yes	116	11	3	27
	Total	1076	100	11	100
Oilseed rape	No	563	92	405	86
	Yes	51	8	68	14
	Total	614	100	473	100
Beet	No	681	90	287	88
	Yes	79	10	40	12
	Total	760	100	327	100
Potato	No	256	83	712	91
	Yes	52	17	67	9
	Total	308	100	779	100
Vegetables	No	79	56	889	94
	Yes	63	44	56	6
	Total	142	100	945	100
Fruit trees	No	40	71	928	90
	Yes	16	29	103	10
	Total	56	100	1031	100
All	No	968	89	–	–
	Yes	119	11	–	–
	Total	1087	100	–	–

Direct interviews were conducted in 15 locations with producers of agricultural, horticultural and special crops. These were farms of various sizes and production types: three large-area farms (450–1400 ha) mainly engaged in the production of agricultural crops; three large-area (600–1100 ha) and three smaller (20–80 ha) farms focused on the production of vegetables; four fruit farms; as well as two farms growing hops.

All methods were carried out in accordance with relevant guidelines and regulations. Informed consent was obtained from all subjects—the questionnaires were anonymous. All respondents were of legal age. None of the experimental protocols required approval by licensing committees or institutions.

## Results and discussion

The survey results indicate that for most farmers and horticulturists in Poland, i.e. for 96.7% of respondents, the knowledge of soil properties, including moisture, is important, although currently only 4.3% of them monitor soil moisture in their farms (Table 4). It follows from this data that farmers are aware of the impact of soil moisture on the conditions and effects of plant cultivation, but they have no possibility to make an ongoing assessment and analysis. Such a situation exists not only in Poland, but also in other countries. Even innovative and well-developed soil moisture monitoring systems on a global scale have limited application in individual farms^23,29^. Farmers expect easy-to-use devices that monitor soil moisture for the purpose of making optimal decisions when irrigating arable crops^30^. Similar expectations of Polish farmers were confirmed by the high percentage of respondents - 83.2%, who declared the willingness to test such devices in their farms prior to purchasing them.

**Table 4:** Assessment of the need and current state of monitoring of soil properties, including soil moisture.

Question	Answer	Number	%
Is knowledge of soil moisture, important?	Yes	1009	96.7
	No	35	3.3
	Total	1044	100
Is soil moisture currently monitored using probes?	Yes	45	4.3
	No	1008	95.7
	Total	1053	100
Declaration of willingness to test/use devices for monitoring of soil properties	Yes	878	83.2
	No	177	16.8
	Total	1055	100

**Table 5:** Dielectric properties of the probes selected for evaluation of soil properties.

Probe	Measurement principle	Independence of soil moisture measurement on		Soil salinity measurement	Soil temperature measurement	Cable connections	Wireless network
		Salinity	Texture				
E-Test TDR/MUX/mpts	TDR	Yes	Yes*	Yes	Yes	Yes	Yes
Acclima True TDR-315	TDR	Yes	Yes*	Yes	Yes	Yes	No
FieldScout TDR 150	TDR	Yes	Yes*	Yes	Yes	Yes	Yes
HYDRA 100 Scout	CAP	No	No**	Yes	Yes	No	Yes
WaterScout SMEC 300	CAP	No	No**	Yes	Yes	Yes	No
Campbell Scientific CS655	TDR-like	Yes	Yes*	Yes	Yes	Yes	No
Decagon 5TE	CAP	No	No**	Yes	Yes	Yes	No
Delta-T ML3 probe	CAP	No	No**	No	Yes	Yes	No

*For most soils, standard calibration provides sufficient measurement accuracy. Individual calibration may be beneficial for soils with a high content of clay fraction or organic matter.

**Producers may supply several calibration functions for various soil types. It is also advised to perform an individual calibration of the probe for the tested soil type to achieve good measurement accuracy.

*TDR* time-domain-reflectometry, measurement of apparent dielectric permittivity. Moisture measurement is virtually independent of soil salinity (in the salinity range of normal agricultural soils), soil texture influence is usually minimal and negligible for most mineral soils in agricultural applications. For most soils, standard calibration provides sufficient measurement accuracy. Individual calibration may be beneficial for soils with a high content of clay fraction or organic matter.

*CAP* measurement of capacitance or impedance of sensor electrodes inserted into soil at a specific frequency, usually not higher than 100 MHz. The lower the measurement frequency that is used the greater is the potential influence of soil salinity and texture. Producers may supply several calibration functions for various soil types. It is also advised to perform an individual calibration of the sensor for the tested soil type to achieve good measurement accuracy.

Our analysis of the available commercial solutions on the market indicates that the currently available monitoring systems are imperfect and difficult in direct use on a farm, e.g. soil moisture measurement is dependent on its salinity and temperature, measuring probes require connections using a cable and have limited wireless connection options available and no battery power supply. Only one of the solutions cited is completely wireless (HYDRA 100 Scout). It uses, however, capacitive probes, whose measurements are affected by a systematic error resulting from the influence of salinity and soil texture (Table 5).

Despite an awareness of the present technical limitations, the majority (88.4%) of respondents declared their willingness to purchase and use one or more probes to assess the moisture, salinity and temperature of the soil in their farms. A condition was that the probe manufacturers eliminate current design imperfections (Fig. 1). In total, the sample of surveyed farmers declared a demand for 2905 probes, and most of them said they would build a system for monitoring soil properties in their farm in the future. The largest groupof the respondents, 31%, declared an interest to purchase only one probe, but 18.7% said two probes and about 10% of respondents declared an interest to purchase 10 or more probes. This number of devices could make it possible to build a measurement network for monitoring soil properties in a farm. The obtained declarations of purchase and use indicates the farmers' desire for simple, easy-to-use devices for assessing soil moisture, salinity and temperature. According to the results of studies conducted by Jury and Vaux^31^ and Regan et al.^32^, such a high demand for soil moisture monitoring systems may also be a result of the growing economic and ecological awareness of agricultural producers. The consumption of water in agricultural production amounts to approximately 75% of the available global freshwater resources, and it is increasing. Therefore, conserving water is a duty of everyone, including farmers regardless of their type and size of production.

Although a statistically significant relationship was found between the surface area of the farm and the declared number of devices for purchase and use, the correlation coefficient r = 0.223 was low (Fig. 2). Therefore, the size of the farm was not a very important premise that farmers followed when declaring their willingness to purchase equipment for the ongoing assessment of soil properties. Such results confirm, therefore, a high awareness of farmers on the impact of soil moisture, salinity and temperature on production efficiency and the environment. This is also indicated by a stronger relationship between the irrigated area within the farm and the declared number of probes to be used. There the correlation coefficient was r = 0.352 (Fig. 3). This is justified because irrigation is a high-cost element of cultivation technology, and its effectiveness depends on the properties of the soil. Therefore, the knowledge of its moisture, but also temperature and salinity, allows one to optimize the time of irrigation, the dose of water and coexisting fertilizing. The spatial variability of soil also contributes to the need for accurate monitoring of soil properties in an irrigated field^33^.

The characteristics of an agricultural holding, i.e. the structure of the cultivated plants and the use of an irrigation system, did not have a major impact on farmers' interest in the use of equipment for an ongoing assessment of soil properties. This interest was high and varied from 65.1 to 84.5%, depending on the farm characteristics (Table 6). In the group of farmers using irrigation systems, as many as 83% indicated the willingness to have an average of 4.15 probes in their farms. Also, in the group of farms not currently using irrigation, the interest in these devices was high with 74% of farmers declaring a potential average use of 3.51 probes. In farms not cultivating cereals, and thus having other intensive crops, the interest in these devices was the highest with an average of 7.5 probes per farm. The data in Table 6 show that a relatively high interest in devices for monitoring soil moisture, salinity and temperature was demonstrated by farmers growing various groups of plants. However, the largest number of such declarations were made by farmers growing vegetables - 84.5%. Farmers growing oilseed rape were also more interested in using probes than those who do not grow oilseed rape. It follows, therefore, that those most interested in the use of these devices were farmers growing crops generating potentially large profits, but requiring favourable soil conditions the correction of which is possible by agricultural practices when the soil moisture, salinity and temperature are known.

**Table 6:** Declaration of demand for devices monitoring soil properties depending on the characteristics of the farm.

Farm element	Occurrence of elements							
	Yes				No			
	Number of respondents	Declaration of use of the device	%	Mean number of probes	Number of respondents	Declaration of use of the device	%	Mean number of probes
Irrigation	119	99	83.2	4.15	968	723	74.7	3.51
Cereals	1076	814	75.7	3.55	11	8	72.7	7.50
Oilseed rape	614	514	83.7	3.63	473	308	65.1	3.51
Beet	760	582	76.6	3.49	327	240	73.4	3.81
Potato	308	231	75.0	3.53	779	591	75.9	3.61
Vegetables	142	120	84.5	3.43	945	702	74.3	3.61
Fruit trees	56	41	73.2	2.68	1031	781	75.8	3.63

The declarations of purchase and use of devices for the assessment of soil properties were regionally diversified (Table 7). All surveyed farmers and horticulturists from the Mazovian voivodeship expressed a need to have such devices in their farms. It should be noted that this is the region of Poland with the largest concentration of orchards and with many vegetable farms. In the West Pomeranian voivodeship, in turn, there are large-scale agricultural farms, whose users have a high awareness of spatial and temporal diversification of soil properties and their impact on the effectiveness of agricultural practices^34^. These conditions were also emphasized by farmers in direct interviews.

**Table 7:** Geographic and administrative diversity of the willingness to use devices for monitoring soil properties.

Voivodeship	Declaration of use			
	Yes		No	
	Number	%	Number	%
Lower Silesian	2	40	3	60
Kuyavian-Pomeranian	446	75	149	25
Lublin	22	85	4	15
Lubusz	13	59	9	41
Łódź	12	75	4	25
Mazovian	8	100	0	0
Pomeranian	83	78	23	22
Warmian-Masurian	7	78	2	22
Greater Poland	187	74	66	26
West Pomeranian	40	98	1	2
Total/mean	820	76	261	24

More than 90% of farmers in the surveys declared that the knowledge of soil moisture, temperature and salinity is helpful in determining the starting date of field works and sowing (Table 8). In their opinion, the influence of soil properties on other cultivation practices, such as fertilization, plant protection and irrigation, is also high. Such reasoning is fully justified, because the soil properties determine its bearing capacity and traction capacity for tractors and agricultural machinery without adversely affecting the soil structure^35,36^. Soil temperature and moisture, in turn, are the basic factors of seed germination and plant growth^37–39^.

**Table 8:** Assessment of the impact of soil properties on the optimization of agrotechnical procedures—farmers' declarations.

Agricultural treatment	Declaration	Number	%
Date of starting field works	No	48	9.2
	Yes	475	90.8
	Total	523	100.0
Sowing date	No	32	6.1
	Yes	490	93.9
	Total	522	100.0
Sowing depth	No	119	24.6
	Yes	365	75.4
	Total	484	100.0
Soil cultivation method	No	90	18.6
	Yes	394	81.4
	Total	484	100.0
Fertilization method/rate	No	127	25.9
	Yes	363	74.1
	Total	490	100.0
Application of plant protection products	No	132	28.0
	Yes	339	72.0
	Total	471	100.0
Irrigation time/dose of water	No	129	40.6
	Yes	189	59.4
	Total	318	100.0
Irrigation time/dose of water*	No	5	17.2
	Yes	24	82.8
	Total	29	100.0

*Only respondents declaring crop irrigation.

Most farmers have recognized that the most important features that characterize a good soil monitoring device are measurement accuracy and reliability. Also important are the ability to assess soil properties at various depths, wireless data transmission and estimation of the water dose during irrigation based on the obtained soil moisture and temperature (Table 9). According to farmers, the price of equipment is also important although it is not the most important.

**Table 9:** Required features of devices for soil properties monitoring according to farmers' expectations.

Device characteristic	Characteristic significance					
	Not important		Important		Very important	
	Number	%	Number	%	Number	%
Low price	73	7.04	526	50.7	438	42.2
Measurement accuracy	28	2.73	386	37.7	611	59.6
Measurement depth 0–15 cm	77	8.38	533	58.0	309	33.6
Depth 0–15 cm and 15–30 cm	88	9.21	523	54.8	344	36.0
Reliability and damage resistance	43	4.37	395	40.2	545	55.4
Easy assembly and service	62	6.13	462	45.7	487	48.2
Wireless data transmission	187	18.9	518	52.5	282	28.6
Estimation of water dosage	134	14.8	464	51.2	308	34.0
Estimation of water dosage*	8	7.84	44	43.1	50	49.0
Irrigation automation*	21	22.1	38	40.0	36	37.9
Irrigation automation	214	24.5	436	49.9	224	25.6

*Only respondents declaring crop irrigation.

Farmers' expectations regarding the performance features of the probes and the possibility of using them in the optimization of agrotechnical treatments were most similar in the Kuyavian-Pomeranian (KP), Greater Poland (WP), and Pomeranian (PM) voivodships. These are regions of Poland with typical family farms conducting commercial crop and livestock production with the inclusion of horticultural production. Farmers from the Lubusz (LU) and West Pomeranian (ZP) voivodships—regions of north-western Poland with large-scale farms focused mainly on field crop production—had the most different expectations for the probes (Fig. 4).

According to the expectations of farmers from their respective voivodeships of Poland, the most important features are the possibility of using the probes to optimize the method and dose of fertilization^5^ and the choice of the date of fertilization of plants^7^, which is not correlated with the aforementioned characteristic—it is the greatest contribution to the first component. According to the respondents, the operational features of the probes are also important, such as their reliability, ease of use, transfer of results to a smartphone/computer, and using the results for irrigation of plants^12–16^—it is the second main component (Fig. 5).

Farmers' expectations regarding the functionality of the probes were related to the size of their farms (Fig. 6). The expectations of the farmers of the smallest farms (up to 20 ha) and large farms (of 800–1000 ha) were the most divergent. The requirements of owners of farms with a smaller area, up to 400 ha, differed from the requirements of farmers running production on farms with a large area.

Farmers expected, above all, that the probes would facilitate the use of plant protection products^6^ and that they would be reliable, easy to use, and reported results online^12–14^—these are the first main component (Fig. 7). Probes should also optimize the choice of the date of commencing field works^1^ and fertilization of plants^5,7^ with the ability to measure soil moisture and salinity both in the 0–15 cm and 0–30 cm layer^9,10^—these are the second main component.

The dendrogram (Fig. 8) shows that farmers' expectations as to the performance of the probes depended on the crops they were growing. The first group were farmers growing agricultural crops such as cereals, beetroot, rape and others, and the second group were potato growers. Horticulturists growing vegetables and fruit trees are separate groups.

Farmers cultivating various groups of plants declared that probes monitoring soil moisture and salinity should help in choosing the optimal date of field works^1^, including the date of sowing^2^, the method of soil cultivation^4^ and the method and dose of fertilization^5^—these are the first main component. In addition, the probes should optimize the fertilization date^7^ and plant irrigation^15,16^ on the basis of information sent to a smartphone/computer^14^—these are the second component (Fig. 9).

Direct interviews conducted with a representative sample of farmers confirmed the results of the survey. Regardless of the production sector (agriculture, horticulture), farm size or region, farmers emphasized the need for ongoing monitoring of soil properties, mainly moisture, temperature and salinity. This knowledge would allow them to optimally use the natural fertility of the soil, increase the efficiency of the use of means of production and make agricultural production more environmentally friendly. In their opinion the monitoring of rainfall and air temperature is no longer sufficient. Agricultural producers in Poland believe that nowadays soil properties should be assessed not only on the farm scale, but also in fields and even in fragments of a field. Creating monitoring systems would allow for rational and precise application of water, mineral fertilizers or plant protection products. It would also be easier to make decisions on scheduling work in fields and in orchards. The interviewees pointed to such solutions already functioning in Poland, but adapted to drought monitoring throughout the whole country^40^. Widespread monitoring of soil properties in the farm, especially of monitoring systems, requires a technical improvement in the devices for assessing soil moisture, salinity and temperature. Jones et al.^41^ indicate a problem that may explain why such a small percentage of farmers use soil moisture probes: the growing number of new sensors across the globe is creating a market filled with confusing choices for consumers and decreasing market share for producers. Without informed consumer choices a product price point may be controlled more by advertising advantage than by product performance and quality. Technical problems connected to the operation and servicing of such equipment are the most frequent reasons for discontinuation of their use by respondents. In the farmers' opinion, these devices must be easy to use and reliable. However, as in drought monitoring^42^, they should contain advanced computer applications for optimizing and verbalizing cultivation recommendations.

## Conclusions

1. Currently, only 4.0% of the surveyed agricultural and horticultural farms use soil moisture, temperature and salinity probes, but as many as 80% of the respondents declare their willingness to purchase and use them. Most of the respondents report the need to purchase one probe for point measurements of soil parameters, but about 10% of respondents declared their willingness to purchase the probes in quantities that would allow for the creation of farmland monitoring systems.
2. The price of the probes in question is not the most important purchase criterion. Farmers and horticulturists expect probes that are easy and practical to use, reliable and durable. Agricultural producers are also interested in applications that facilitate making agrotechnical decisions.
3. The obtained results prove the insufficient supply of probes meeting the expectations of agricultural producers and show a high demand for equipment with appropriate utility features. The analysis of currently available solutions shows that the market does not offer such devices. This is also confirmed by the opinions of the respondents.
4. The vast majority of farmers are aware of the importance of assessing soil moisture, temperature and salinity in optimizing irrigation, fertilization and pesticide application as well as other agrotechnical procedures.
5. Farmers using irrigation systems, as well as producers of vegetables and rape, showed a greater interest in purchasing probes. A territorial differentiation in this respect was also shown.
6. The presented results could be used by the manufacturers of soil moisture, salinity and temperature probes in order to provide devices more suited to the farmers’ needs and expectations and to adjust their marketing strategies.
